# A High Level of Circulating Valine Is a Biomarker for Type 2 Diabetes and Associated with the Hypoglycemic Effect of Sitagliptin

**DOI:** 10.1155/2019/8247019

**Published:** 2019-11-11

**Authors:** Xiaoyu Liao, Bingyao Liu, Hua Qu, LinLin Zhang, Yongling Lu, Yong Xu, Zhaohui Lyu, Hongting Zheng

**Affiliations:** ^1^Department of Endocrinology, Translational Research Key Laboratory for Diabetes, Xinqiao Hospital, Army Medical University (Former Name: Third Military Medical University), Chongqing, China; ^2^Medical Research Center, Southwest Hospital, Army Medical University (Former Name: Third Military Medical University), Chongqing, China; ^3^Department of Diabetes and Endocrinology, Affiliated Hospital of Southwest Medical University, Sichuan, China; ^4^The Department and Key Laboratory of Endocrinology and Metabolism, The First Medical Center of PLA General Hospital, Beijing 100853, China

## Abstract

**Background:**

High levels of branched-chain amino acids (BCAAs) and aromatic amino acids (AAAs) were associated with an increased risk of hyperglycemia and the onset of diabetes. This study is aimed at assessing circulating valine concentrations in subjects with type 2 diabetes (T2D) and in T2D patients and high-fat diet- (HFD-) fed mice treated with the hypoglycemic agent sitagliptin (Sit) and analyzing the association of valine concentrations with metabolic parameters.

**Methods:**

Metabolomics in HFD-fed mice were analyzed by gas chromatography-mass spectrometry (GC-MS) systems. Plasma valine concentrations were detected with a commercial kit in 53 subjects with normal glucose levels (*n* = 19), newly diagnosed T2D (*n* = 20), placebo-treated T2D (*n* = 7), or Sit-treated T2D (*n* = 7). Biochemical parameters were also assessed in all participants.

**Results:**

Sit treatment markedly changed the pattern of amino acid in HFD-fed mice, especially by reducing the level of the BCAA valine. Compared with the healthy controls, the plasma valine concentrations were significantly higher in the T2D patients (*p* < 0.05). Correlation analysis showed that the plasma valine concentration was positively correlated with the level of fasting plasma glucose (*p* < 0.05). Moreover, the plasma valine concentrations were notably reduced after Sit treatment in T2D patients (*p* < 0.05).

**Conclusions:**

Our findings demonstrate an important effect of Sit on the BCAA valine in T2D patients and HFD-fed mice, revealing a new hypoglycemic mechanism of it. Furthermore, the results suggest that the circulating valine level might be a novel biomarker for T2D and restoring the level of valine might be a potential strategy for diabetes therapy.

## 1. Introduction

The intestinal gut microbiota is an intricate ecosystem. Existing evidence has indicated that the gut microbiota is involved in regulating the homeostasis of the host metabolism [1]. The dysbiosis of the gut microbiota is associated with metabolic diseases, including diabetes [2], obesity [3, 4], and insulin resistance [5]. Although it is now commonly accepted that the microbiome is closely related with glucose metabolism, the molecular mechanism revealing the regulation of glucose metabolism by bacteria is still not clear. Emerging evidence has suggested that short chain fatty acids (SCFAs), bile acids, and amino acids may be the potential mechanism [6–8].

Our recent study reported that the commonly used oral hypoglycemic agent, dipeptidyl peptidase-4 inhibitors (DPP-4i), markedly changed the composition of the gut microbiota and the pattern of metabolites in high-fat diet- (HFD-) fed mice and that the DPP-4i sitagliptin- (Sit-) altered microbiome from type 2 diabetes (T2D) patients improved the glucose intolerance induced by HFD in germ-free mice [9]. Sit treatment resulted in a trend toward an increase in SCFAs, but with no significance. Therefore, we set out to focus on the alteration of other metabolites, such as amino acids, to explain the functional mechanism of DPP-4i-altered microbiome in modulating the glucose metabolism in the further study.

Accumulating studies have indicated that obese individuals have higher concentrations of branched-chain amino acids (BCAAs) (isoleucine, leucine, and valine) and aromatic amino acids (AAAs) (phenylalanine and tyrosine), and these amino acids have been correlated with blood glucose and insulin levels [10–13]. In addition, it has been reported that BCAA and AAA levels are associated with the extent of insulin resistance [14–19]. Moreover, a prospective study found that fasting concentrations of amino acids were elevated up to 12 years prior to the onset of diabetes and that the risk of diabetes increased at least 4-fold among individuals with high concentrations of plasma amino acids [12]. Recently, it has been reported that the classic hypoglycemic agent metformin could change the pattern of amino acids in randomized clinical trials [20, 21]. Based on these studies, amino acids might be associated with the improvement of glucose metabolism modulated by DPP-4i-altered microbiome.

In the current study, we analyzed the alteration of amino acids in the Sit-treated HFD-fed mice, and further analyzed the alteration of valine induced by Sit treatment in HFD-fed mice and newly diagnosed T2D patients, and analyzed the correlations between circulating valine levels and metabolic parameters among study participants.

## 2. Subjects and Methods

### 2.1. Study Subjects

#### 2.1.1. T2D Patients and Healthy Controls

Thirty-nine individuals were recruited for our study. The T2D patients were newly diagnosed based on the World Health Organization 1998 diagnostic criteria [22]: fasting plasma glucose (FPG) ≥ 7.0 mmol/L, 2-hour postprandial plasma glucose (2hPG) ≥ 11.1 mmol/L, or both. The exclusion criteria were as follows: (i) pregnancy, (ii) smoking and alcohol consumption history, (iii) presence of acute or chronic complications of diabetes, and (iv) presence of gastrointestinal disorders or a history of chronic physical/mental diseases, such as Alzheimer's disease or Parkinson's disease. The experimental protocol was approved by the Ethics Committee of Xinqiao Hospital, Third Military Medical University, and registered online (Clinical trial registry number ChiCTR-ROC-17010719).

#### 2.1.2. Sitagliptin-Treated Subjects

Briefly, fourteen newly diagnosed T2D participants were randomized into two groups: one group was treated with sitagliptin (Sit; *n* = 7), and the other group was treated with placebo (*n* = 7). Sit (Merck Sharp & Dohme, USA) was administered at a dose of 100 mg/d. Moreover, all individuals were recommended to maintain a reduced daily caloric intake of 25 kcal/kg and perform regular physical exercise (2.5 hours/week) throughout the entire study [23]. The detailed inclusion and exclusion criteria for T2D patients were described in our previous study [9]. Informed written consent was obtained from all participants. The experiment was approved by the Ethics Committee of Xinqiao Hospital, Third Military Medical University. The clinical trial registration is deposited in the Chinese Clinical Trial Registry (ChiCTR-OPC-17010757).

### 2.2. Animal Study

C57BL/6 male mice were purchased from the Model Animal Research Center of Nanjing University and were fed a high fat diet (HFD; 60% fat, 20% protein, 20% carbohydrate (kcal/100 g), D12492; Research Diets, New Jersey, USA) for 14 weeks. Then, the mice were divided into different groups: Sit-treated group and control group. The Sit-treated group was administered 4 g/kg of Sit mixed with HFD for 4 weeks, and the controls were only fed a HFD during the experiment. The metabolites were extracted from fecal samples according to the manufacturer's instructions (Majorbio Bio-Pharm Technology Co., Ltd., Shanghai, China) and assayed using a 7890A-5975C gas chromatography-mass spectrometry (GC-MS) detection system (Agilent Technologies, Santa Clara, CA). The detailed protocol of the animal study was described in our previous study [9].

### 2.3. Measurement of Plasma Valine Levels

Plasma valine levels were measured by a commercial enzyme-linked immunosorbent assay kit according to the manufacturer's instructions (General Valine ELISA kit, catalog no: E2143Ge, EIAab Science Co. Ltd., China). The detection range of the kit was 1.56~100 nmol/mL. The intra-assay coefficient of variation was 4.6%, and the interassay coefficient of variation was 7.1%. All plasma samples were diluted 20 times before detection. There was no significant cross-reactivity or interference throughout the assay.

### 2.4. Statistical Analyses

The significance of differences between two groups was evaluated using Student's *t*-test. The amino acid concentrations of the HFD-fed mice were numerically transformed before heat map analysis. The correlations between plasma valine levels and metabolic characteristics were estimated using the Pearson correlation coefficient. A *p* value < 0.05 was defined as statistically significant. The statistical analyses were performed with SPSS software version 16.0 (IBM, Armonk, NY).

## 3. Results

### 3.1. Sitagliptin Treatment Reduces the Valine Level in HFD-Fed Mice

**Figure 1:**
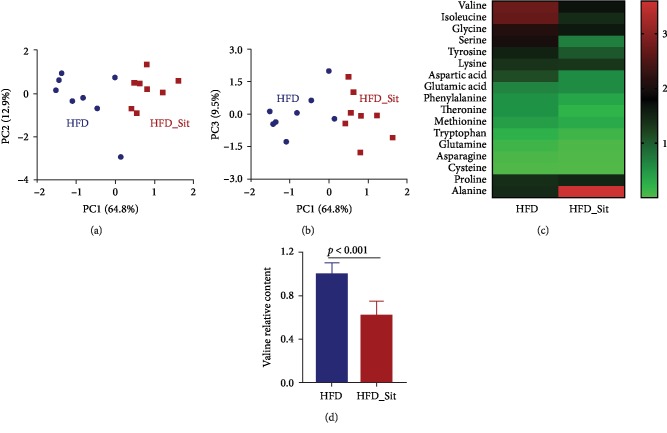
Sitagliptin treatment reduced the valine levels in HFD-fed mice. (a, b) Cluster analysis of the HFD-fed and HFD_Sit mice based on the amino acid concentrations using PCA. The principal components (PC1, PC2, and PC3) from the PCA are plotted for each sample. The percentage of variation covered in the plotted principal components is marked on the axes. Each group is labeled by a different symbol, and each spot represents one sample. (c) Heat map analysis of amino acid levels in the HFD and HFD_Sit groups. Each column in the heat map represents the group, and each row represents one amino acid. The color bar showing green to red indicates the relative level of each amino acid. (d) The relative level of valine in the HFD and HFD_Sit groups. (a–d) *n* = 8; data in (d) are presented as the means ± SD, *t*-test.

In a recent study, we demonstrated that metabolites were markedly changed after Sit treatment in HFD-fed mice [9]. To further explore the alterations of amino acids induced by Sit treatment in HFD-fed mice, principal component analysis (PCA) and heat map analysis were performed based on the amino acid concentrations detected by GC-MS. As shown in Figures 1(a) and 1(b), there was a difference in the distribution of the amino acids between the Sit-treated (HFD_Sit) and HFD groups. In addition, most amino acids presented a decreasing trend in the HFD_Sit mice (Figure 1(c)). Among these amino acids, the relative concentration of valine was the highest and was significantly reduced after Sit treatment (Figure 1(d)). These results suggested that Sit promoted a pattern shift of amino acids and that amino acids, especially valine, might contribute to the hypoglycemic effect of Sit.

### 3.2. The Plasma Valine Level Was Higher in T2D Patients

To observe whether the concentration of valine is associated with diabetes, a total of 20 newly diagnosed T2D patients and 19 healthy controls were enrolled in this study. The main clinical characteristics of the groups with healthy or T2D individuals are shown in Table 1. There were no significant differences in sex, age, BMI, insulin, total cholesterol (TC), or low-density lipoprotein cholesterol (LDL-C) between the two groups. In comparison with the healthy controls, the T2D patients had higher HbA1c, fasting plasma glucose (FPG), HOMA-IR, and triglyceride levels (*p* < 0.001, *p* < 0.001, *p* < 0.001, and *p* = 0.037, respectively), while the levels of HOMA-*β* and HDL-C were significantly lower in the T2D group (*p* = 0.010 and *p* = 0.018, respectively).

**Figure 2:**
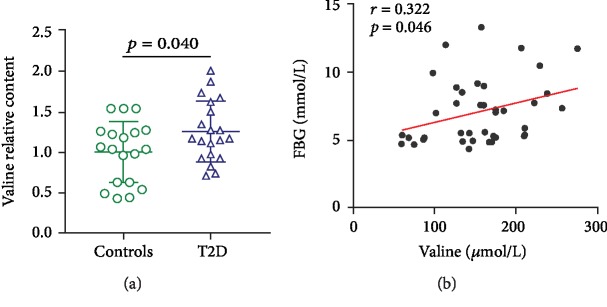
The plasma valine level was higher in T2D patients. (a) The plasma level of valine in healthy controls (*n* = 19) and T2D patients (*n* = 20). (b) The correlation between plasma valine level and fasting plasma glucose concentration (*n* = 39). The data in (a) are presented as the means ± SD, *t*-test.

As expected, when compared to healthy controls, the T2D group displayed a significant increase in plasma valine levels (Table 1, Figure 2(a), *p* = 0.040). In addition, we analyzed the association between valine concentration and metabolic parameters. As shown in Table 2 and Figure 2(b), the plasma valine levels were positively associated with fasting plasma glucose (*p* = 0.046, *r* = 0.322). After adjusting for age and sex, the correlation remained significant (*p* < 0.05). The correlations between valine concentrations and other parameters, such as insulin and HbA1c, were not statistically significant, which might be due to the small sample size.

### 3.3. Sitagliptin Treatment Decreased the Plasma Valine Level in T2D Patients

**Figure 3:**
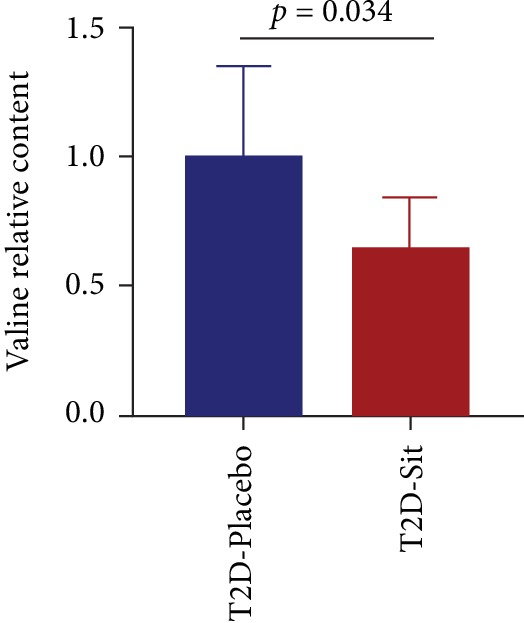
Sitagliptin treatment decreased valine levels in T2D patients. The relative level of valine in the T2D_Placebo and T2D_Sit groups. Data are presented as the means ± SD, *t*-test.

Next, to further investigate whether the hypoglycemic agent DPP-4i could change the valine concentration in the clinical setting, we detected the plasma valine level in another subset of T2D patients who were either treated with Sit or not treated with any antidiabetic agent. This subset of individuals was enrolled in our previous study [9]. As shown in Figure 3, the plasma valine level decreased notably after Sit monotherapy (*p* = 0.034). Taken together, these results demonstrated that valine levels were higher in T2D patients and were decreased after Sit treatment. It suggests that valine might be a potential biomarker for T2D and restoring the level of valine might be a potential strategy for diabetes therapy.

## 4. Discussion

High circulating concentrations of BCAAs (isoleucine, leucine, and valine) and AAAs (phenylalanine and tyrosine) have recently been shown to be associated with an increased risk of hyperglycemia and the onset of diabetes [12, 13, 24]. Valine (2-amino-3-methylbutyric acid), a BCAA, is an essential amino acid in humans. To our knowledge, this is the first study to analyze valine levels in T2D patients and HFD-fed mice after Sit treatment. We observed that Sit treatment markedly changed the pattern of amino acids and decreased the valine level in HFD-fed mice. Furthermore, compared with the healthy controls, the valine level was higher in T2D patients, while it decreased after Sit monotherapy. These results suggest that valine might be involved in the pathogenesis of T2D and might be related to the hypoglycemic therapy for T2D. It also demonstrates a novel effect of DPP-4i on the BCAAs, which might be a new hypoglycemic mechanism of this drug.

The gut microbes act as bioreactors that ferment dietary components to produce health-promoting metabolites, such as amino acids [25–27], short-chain fatty acids (SCFAs) [28, 29], and vitamins [30, 31]. Essential amino acids (e.g., arginine and BCAAs) cannot be synthesized by humans; these essential amino acids originate from dietary components that are metabolized by microbes in the ileum [32]. The concentration of amino acids is partially correlated with the structure of microbial community [33]. In an analysis of the host metabolism and gut microbiota in individuals with insulin resistance, increased levels of BCAAs were correlated with the abundance of the gut microbiota, especially the abundance of *Prevotella copri* and *Bacteroides vulgatus*. These two kinds of bacteria were identified as the main species with the potential to biosynthesize BCAAs. *P. copri* could increase the serum levels of BCAAs in HFD-fed mice [5]. Thus, the alterations of gut microbial composition in the context of diabetes might be an important reason for the increase in BCAAs.

The altered BCAAs seem to be related to the specific intestinal flora community in diabetes, while the composition of gut microbiota was found to be influenced by antidiabetic therapy, especially hypoglycemic agents. Therefore, there may be a potential link between BCAAs and antidiabetic therapy. A previous study found that BCAA levels decreased rapidly following glipizide treatment in T2D patients [24]. In addition, metformin also tended to reduce BCAA concentrations, although the change was not statistically significant [21]. In our study, the concentrations of BCAAs were also presented decreasing trend in the Sit-treated HFD-fed mice, and the level of BCAA valine decreased significantly after Sit treatment in newly diagnosed T2D patients and HFD-fed mice. Taken together, these studies demonstrated that BCAAs might be a potential biomarker for diabetes and that restoring circulating levels of BCAAs might be a novel strategy for diabetes therapy. Our results also suggest that the reduction of BCAA valine might be one of the mechanisms of improving glucose metabolism by DPP-4i.

A prospective observational study reported that the elevation of BCAAs is associated with the risk of T2D [12, 34], and recent genetic studies have suggested the important role of BCAA metabolism in the development of diabetes [35]. The molecular mechanism of the involvement of BCAAs in the pathogenesis of diabetes is still unclear. Branched-chain alpha-ketoacid dehydrogenase (BCKD), responsible for the rate-limiting step of BCAA catabolism, is markedly lower in individuals with T2D [36, 37]. In addition, BCKD could be activated by its regulatory phosphatase PPM1K, and the expression of PPM1K was also downregulated in individuals with T2D [38]. It has also been reported that BCAAs are related to insulin resistance [14, 39]. Moreover, 3-hydroxyisobutyrate (3-HIB), a catabolic intermediate of valine, is viewed as a link between BCAA catabolism and insulin resistance. 3-HIB, as a new paracrine regulator of transendothelial fatty acid transport, could also activate transendothelial fatty acid transport, promote fatty acid uptake and accumulation in the muscle, and lead to insulin resistance [40]. In addition, BCAAs have been shown to modulate insulin secretion, except for insulin resistance [41, 42], and another possible mechanism by which elevated BCAAs promote diabetes is via hyperinsulinemia leading to pancreatic beta cell dysfunction [12]. However, some studies support the idea that elevated BCAAs are the result of insulin resistance, not the cause of it [43]. The role and functional mechanism of BCAAs, especially valine, in glucose metabolism requires further investigation.

The current study has some limitations that require emphasis. First, our study used a cross-sectional design, so the causality between valine levels and T2D cannot be clarified. Second, as the sample size for detecting valine levels in T2D patients was small, the correlations between valine levels and various metabolic parameters were not statistically significant. Therefore, clinical studies with larger sample sizes should be performed to confirm the correlation between valine levels, the progression of T2D, and the hypoglycemic effect of DPP-4i. Further molecular experiments should be performed to explore the molecular mechanism of valine in regulating glucose metabolism.

In conclusion, our study demonstrated that hypoglycemic agent DPP-4i treatment markedly changed the pattern of amino acids in HFD-fed mice, especially by reducing the level of the BCAA valine. Moreover, the circulating valine levels were significantly higher in T2D patients and were decreased by DPP-4i treatment. The plasma valine concentrations were closely correlated with fasting plasma glucose. Thus, the circulating valine level might be a biomarker for T2D and might be a potential therapeutic target for T2D.

## Figures and Tables

**Table 1:** Clinical characteristics of enrolled healthy and T2D individuals.

	Health (*n* = 19)	T2D (*n* = 20)	*p* value
Sex (M/F, *n*/*n*)	7/12	11/9	0.341
Age (years)	48.58 ± 3.19	51.90 ± 3.18	0.466
BMI (kg/m^2^)	23.15 ± 0.81	25.04 ± 0.89	0.125
HbA1c (%, mmol/mol)	5.66 ± 0.06	8.31 ± 0.50	<0.001
FPG (mmol/L)	5.14 ± 0.09	9.00 ± 0.43	<0.001
2hPG (mmol/L)	4.64 ± 0.12	9.75 ± 0.56	<0.001
Insulin (mU/L)	6.37 ± 1.27	8.80 ± 1.02	0.144
HOMA-IR	1.47 ± 0.31	3.44 ± 0.40	<0.001
HOMA-*β*	78.92 ± 14.71	36.82 ± 5.96	0.010
Total cholesterol (mmol/L)	4.75 ± 0.19	4.60 ± 0.20	0.586
Triglycerides (mmol/L)	1.26 ± 0.15	1.96 ± 0.28	0.037
HDL-C (mmol/L)	1.59 ± 0.12	1.22 ± 0.09	0.018
LDL-C (mmol/L)	2.70 ± 0.19	2.77 ± 0.21	0.824
Valine (*μ*mol/L)	137.56 ± 11.77	172.59 ± 11.48	0.040

BMI, body mass index; FPG, fasting plasma glucose; 2hPG, 2-hour postprandial plasma glucose; HOMA-IR, homeostasis model assessment for insulin resistance; HOMA-*β*, homeostasis model assessment for beta-cell function; HDL-C, high-density lipoprotein cholesterol; LDL-C, low-density lipoprotein cholesterol. Data are presented as means ± SEM. *p* value was calculated by *t*-test.

**Table 2:** Correlation coefficient of clinical characteristics associated with plasma valine concentration in the study individuals.

	Plasma valine		Plasma valine (age- and sex-adjusted)	
	*r*	*p* value	*r*	*p* value
Age (year)	0.008	0.963	—	—
Sex (M/F)	0.133	0.421	—	—
BMI (kg/m^2^)	-0.225	0.168	-0.210	0.212
HbA1c (%, mmol/mol)	0.231	0.156	0.273	0.103
FPG (mmol/L)	0.322	0.046	0.340	0.040
2hPG (mmol/L)	0.277	0.088	0.301	0.071
Insulin (mU/L)	-0.123	0.456	-0.126	0.457
HOMA-IR	-0.026	0.873	-0.027	0.873
HOMA-*β*	-0.279	0.085	-0.299	0.073
Total cholesterol (mmol/L)	0.069	0.674	0.076	0.654
Triglycerides (mmol/L)	0.063	0.703	0.070	0.679
HDL-C (mmol/L)	0.019	0.907	0.002	0.989
LDL-C (mmol/L)	0.054	0.743	0.074	0.664

BMI, body mass index; FPG, fasting plasma glucose; 2hPG, 2-hour postprandial plasma glucose; HOMA-IR, homeostasis model assessment for insulin resistance; HOMA-*β*, homeostasis model assessment for beta-cell function; HDL-C, high-density lipoprotein cholesterol; LDL-C, low-density lipoprotein cholesterol. Correlations between valine concentration and clinical characteristics were analyzed by Pearson's analysis or an age- and sex-adjusted partial correlation test, while the correlation between valine concentration and sex was analyzed by Spearman's analysis.

## Data Availability

The metabolomic data used to support the findings of this study have been included in the corresponding author's (Hongting Zheng) published paper (DOI: 10.1016/j.ebiom.2019.03.057).
